# Evaluation of retinal microvasculature in exotropia with abnormal binocular vision by optical coherence tomography angiography

**DOI:** 10.1186/s12886-023-02900-w

**Published:** 2023-04-11

**Authors:** Chun-Wen Chen, Qin-Zhu, Chun-Hui Ma, Jing-Yan Yao

**Affiliations:** Department of Ophthalmology, The First Affiliated Hospital of Soochow University, Suzhou, China

**Keywords:** Large-angle concomitant exotropia, Optical coherence tomography angiography, Macular microvasculature, Binocular vision

## Abstract

**Background:**

To explore the retinal microvasculature in large-angle concomitant exotropia patients with abnormal binocular vision using optical coherence tomography angiography (OCTA) analysis.

**Methods:**

OCTA images of 52 healthy and 100 strabismic eyes were analyzed to quantify the retinal thickness (RT), superficial capillary plexus (SCP), deep capillary plexus (DCP), and foveal avascular zone (FAZ). Paired t-tests were performed to compare differences between the two groups, the dominant eye and the deviated eye in the exotropia group, respectively. A *p*-value < 0.01 was considered significant.

**Results:**

The mean angle of deviation was 79.38 [± 25.64] (prism diopters, PD). There were significant differences in the DCP in deviated eyes between the exotropia group and the control group (fovea: *p* = 0.007; temporal: *p* = 0.014; nasal: *p* = 0.028; inferior: *p* = 0.013). The temporal SCP in the exotropia group was significantly higher than in the control group in deviated eyes (*p* = 0.020). No significant difference was found between dominant eyes and strabismic eyes (*p* > 0.01).

**Conclusions:**

The study showed that OCTA revealed subnormal DCP in patients with large-angle exotropia and abnormal binocularity which may be related to retinal suppression. Changes in the macular microvasculature may provide valuable insights into the development of strabismus. Further studies are needed to determine the clinical relevance of this finding.

**Trial registration:**

This trial is registered as ChiCTR2100052577 at www.Chictr.org.cn.

## Background

Concomitant exotropia is described as manifest divergent strabismus, defined as the angle independent from the direction of gaze [1]. It affects 1% to 4.2% of the child population [2]. It mainly manifests loss of binocularity and stereopsis. In addition to binocular dysfunction, patients with concomitant exotropia experience psychological distress, anxiety, and depression [3, 4]. In general, patients with large-angle constant exotropia are prone to fatigue, amblyopia, and impaired binocular visual function to maintain alignment [5, 6]. The mechanism of constant exotropia is not well understood.

Optical Coherence Tomography Angiography (OCTA) is a non-invasive and effective fundoscopic examination technology to perform the visualization of blood flow in the retina. It has been widely used in many ophthalmic diseases such as glaucoma, age-related macular degeneration, diabetic retinopathy, amblyopia, and myopia [7, 8]. OCTA has provided novel insights into the pathophysiology of various retinal diseases, including the perfusion of not only the superficial capillary plexus (SCP) and deep capillary plexus (DCP), but also the choriocapillaris [9, 10]. Previous studies have shown that early retinal and choroidal capillary defects in amblyopia and myopia may be essential for monitoring the disease [11–14]. Several studies found inconsistent changes in macular structure in the strabismic eye, including increased central macular thickness postoperatively and decreased total macular thickness in the dominant eye, although these pathoanatomical findings have not been confirmed [15, 16].

Mintz HR et al. [15] found a subclinical increase in macular thickness after extraocular muscle surgery. It may be related to the new position of the extraocular muscles, postoperative inflammation, and changes in the blood-retinal barrier. Oka M et al. [16] found that strabismic patients with abnormal binocular vision development had thinner superior temporal GCC thickness in the dominant eye. Retinal ganglion cells in this region may contribute to efferent neuronal degeneration in the visual pathway responsible for adaptation to visual experience in patients with concomitant exotropia.

However, macular OCTA findings in large-angle exotropia with abnormal binocular vision have not been reported. The study aimed to evaluate the macular microvasculature and FAZ in patients with constant exotropia and abnormal binocular vision using OCTA.

## Methods

### Patient and public involvement

This retrospective study was conducted at the First Affiliated Hospital of Soochow University between October 2020 and November 2021. The study was approved by the Ethics Committee of the First Affiliated Hospital of Soochow University and adhered to the Declaration of Helsinki. Informed parental consent was obtained for all participants. Patients with concomitant exotropia of ≥ 45 prism diopters (PD) and over 6 years of age were included in the study. The binocular function was measured with the Synoptophore at three levels. The abnormal binocular function was defined as the absence of all grades. Those with high myopia (≤ -6.0 D), hyperopia, anisometropia (≤ -2.50 D), amblyopia, oblique muscle overaction, paralytic strabismus, restrictive strabismus, previous strabismus surgery, or organic eye disease were excluded. Controls were required to have normal binocular vision and a complete absence of any other type of strabismus. Patients with chronic systemic diseases and neurological disorders were also excluded. In total, 50 patients and 26 controls were recruited finally. We compared the difference between the exotropia and the control group. We also made a comparison between the dominant eye and the deviated eye in the exotropia group. The deviated eye was identified as the eye that was always fixated. We chose the right eye of the control group as the dominant eye. All patients underwent a complete ophthalmic examination, including best-corrected visual acuity (BCVA), refraction (Topcon; Tokyo, Japan), prism alternating cover test with accommodative targets for fixation (6 m), ocular motility tests, slit-lamp, posterior segment examination, axial length (IOL Master 700; Carl Zeiss Meditec AG, Jena, Germany) and OCTA. The ability to control exotropia was assessed using the Newcastle Control Score (NCS). Those with a high (poor) NCS (⩾ 4) were identified [17]. Ocular deviations in patients with concomitant exotropia were measured after 45 min of patch application. Refractive errors were analyzed as spherical equivalent values.

**Fig. 1:**
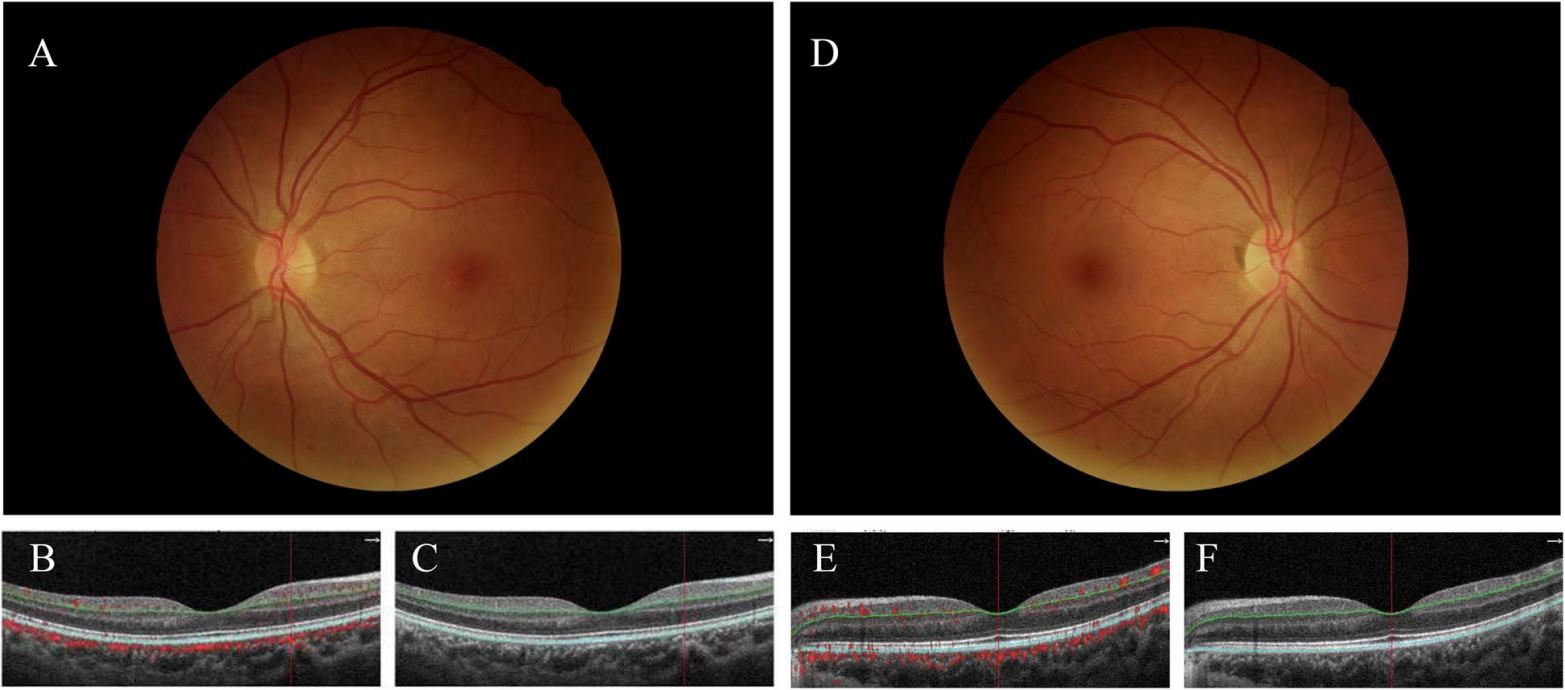
Fundus photographs, optical coherence tomography (OCT), and OCT angiography (OCTA) images analysis of constant exotropia. **A** Fundus photographic image in the deviated eye. **B** OCT image of the SCP in the B-scan image. **C** OCTA image of SCP area in the B-scan image. **D** Fundus photographic image in the dominant eye. **E** OCT image of the DCP in the B-scan image. **F** OCTA image of DCP with in the B-scan image

All participants underwent OCTA imaging with an undilated pupil. The OCTA images were acquired using a spectral-domain device with software (RTVue-XR Avanti with AngioVue software, OptoVue, Inc., Fremont, CA, USA). A 6*6-mm macular scan was performed on each patient. Split-spectrum amplitude-decorrelation angiography was used to examine the OCTA information. Each scan was automatically segmented to visualize the retinal thickness (RT), SCP, DCP of the retina, and FAZ using AngioVue [18]. The representative fundus photographs, OCT, and OCTA images from the deviated eyes and the dominant eyes are shown in Fig. 1.

The middle and outer rings centered on the fovea were divided into four quadrants: superior, nasal, inferior, and temporal. The SCP and DCP vessel density of each region was calculated from the binarised image, using the Otsu method [19], as the percentage of the area defined as the perfusion area [10] over the total area. The SCP en face images were segmented, with an inner boundary below the internal limiting membrane and an outer boundary at 9 μm above the inner plexiform layer. The DCP en face images were segmented with an inner boundary 9 μm above the inner plexiform layer and an outer boundary 15 μm below the outer plexiform layer. FAZ parameters were measured in the 1*1-mm scan. Images with the signal strength of less than 7, or unclear boundaries were excluded. All spectral-domain OCT examinations were performed by a single experienced technician. The technician was blinded to the baseline information of the patients. The OCTA images were included in the hospital’s medical records.

### Statistical analysis

Statistical analyses were performed using SPSS software version 26.0 (SPSS, Inc., an IBM company, Chicago, IL, USA). Values were presented as mean (standard deviation, SD) for normally distributed data or as a median and interquartile range for non-normally distributed data for continuous variables. The normality of the data was tested using the Shapiro–Wilk normality test. Differences between and within groups were determined by analysis of variance (ANOVA) using the least significant difference (LSD) method. The independent samples t-test was used to compare the experimental group with the control group. A paired t-test was used to compare the dominant eye with the deviated eye in the exotropia group. For all analyses, a two-tailed *p-*value < 0.01 was considered statistically significant.

## Results

Demographic characteristics are summarised in Table 1. A total of 100 eyes from 50 consecutive exotropia patients and 52 eyes of 26 healthy subjects were included in this study. The exotropia patients had anomalous retinal correspondence. The sample included 37 males (48.7%). The mean age of the exotropia patients was 24.80 (10.81) years. The angle strabismic patients had abnormal binocular vision and NCS ≥ 4. The mean age of onset of exotropia was 12.97 (2.38) years. The angle of deviation at the distance was 79.38 (25.64) PD. The axial lengths (AL) were 24.38 (1.26) mm and 24.32 (1.12) mm, respectively. No statistically significant difference was found for spherical equivalent, AL, age, and sex (*p* > 0.05).

**Table 1:** Demographic characteristics of patients

Variables	Exotropia group (*n* = 50)	Control group (*n* = 26)	*P* Value
Age, yrs	24.80 (10.81)	22.31 (8.38)	0.309
Sex, No, %			
Male	28 (56)	9 (34)	0.079
Female	22 (44)	17 (66)	
Angle of deviation, PD			
Distance	79.38 (25.64)	0	**< 0.001**
Refraction, D			
Dominant eye	-2.46 (1.53)	-2.67 (1.51)	0.563
Deviation eye	-1.95 (1.93)	-1.75 (1.32)	0.641
Axial length, mm			
Dominant eye	24.40 (1.14)	24.66 (1.04)	0.337
Deviation eye	24.38 (1.26)	24.32 (1.12)	0.855

Data were expressed as mean (standard deviation)

*SD* Standard deviation, *yrs* years, *D* Diopter, *PD* Prism diopters

**Fig. 2:**
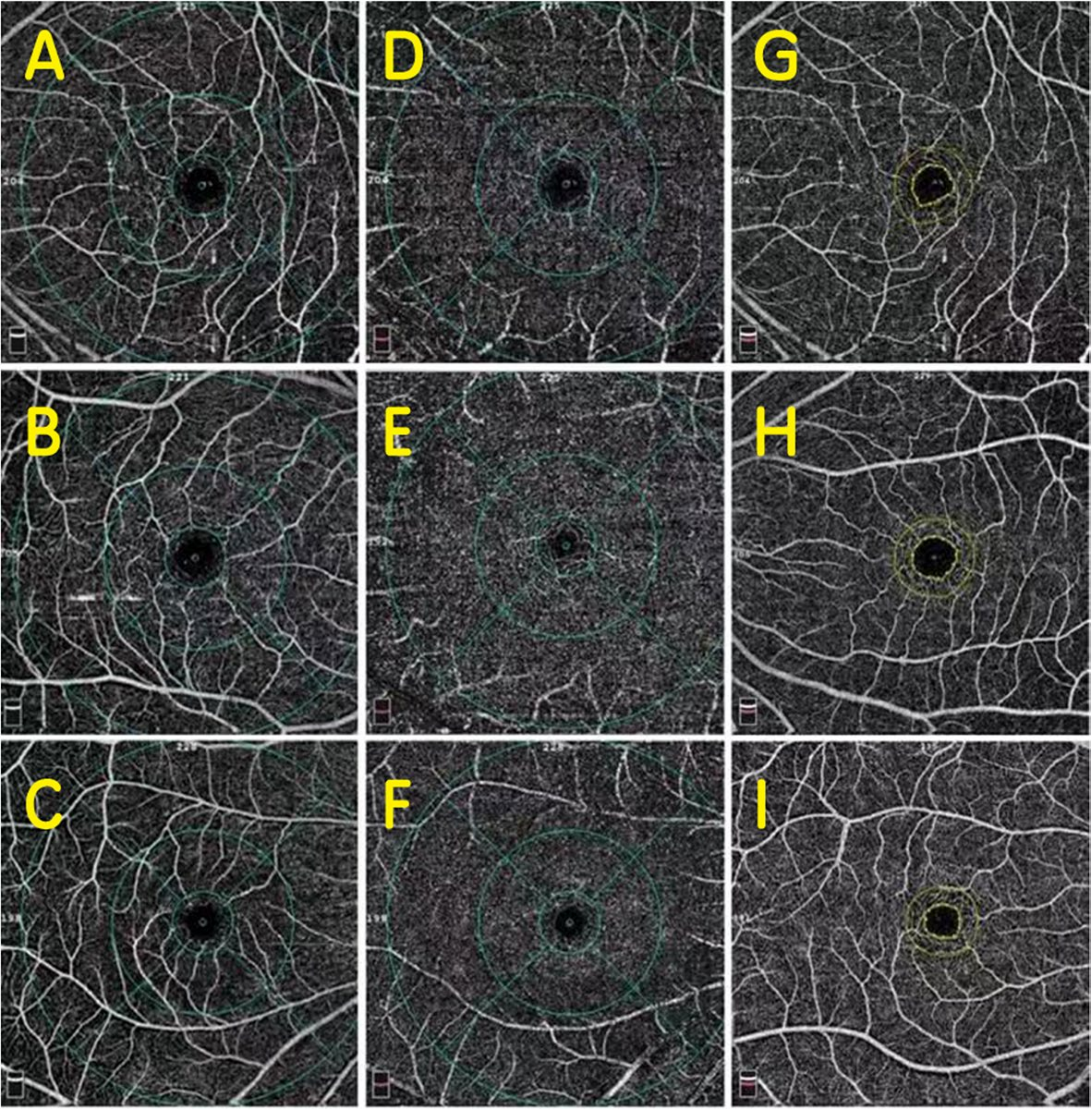
OCTA representative image of the perimeter vessel density and the FAZ area in various quadrants. **A**, **B** and **C** are SCP measurement. **D**, **E** and** F** are DCP measurement. **G**, **H** and **I** are FAZ measurement. **A**, **D** and **G** are deviated eyes. **B**, **E** and **H** are contralateral eyes. **C**, **F** and **I** are control eyes. OCTA, optical coherence tomography angiography; SCP, superficial capillary plexus; DCP, deep capillary plexus; FAZ, foveal avascular zone

**Fig. 3:**
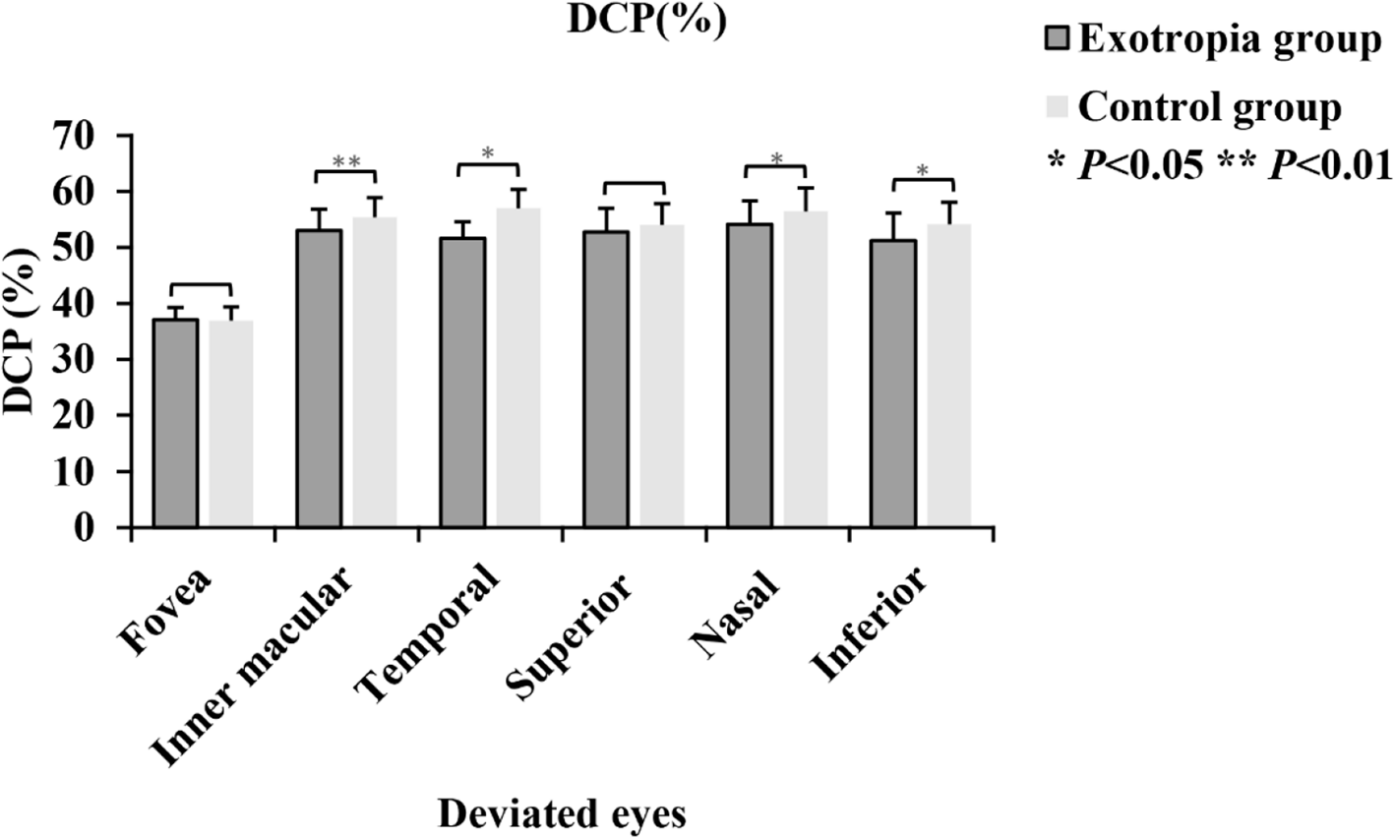
Results of DCP in deviated eyes of foveal and parafoveal regions. Comparison of DCP between the exotropia group and the control group.** Inner macular *p* = 0.007; *Temporal *p* = 0.014; Nasal *p* = 0.028; Inferior *p* = 0.013; Superior *p* = 0.211

OCTA visualization was performed for all metrics (Fig. 2). Macular vessel density and retinal thickness are shown in Table 2. There was no difference in retinal thickness between the deviated eye and the dominant eye (*p* > 0.01). Interestingly, the SCP was greater in the temporal quadrant for dominant eyes in the outer ring (46.80% [3.10%] vs 45.0% [3.00%], *p* = 0.02). For deviated eyes, there were no significant differences in SCP for the exotropia group (*p* > 0.01). The exotropia group had lower DCP in the inner macular area (52.98% [3.85%] vs 55.38% [3.51%], *p* = 0.007) as shown in Fig. 3. There was no difference between the two groups in any area of the FAZ (*p* > 0.01).

**Table 2:** Macular vessel density and retinal thickness between the exotropia group and control group

	Deviated eye		*P* Value	Dominant eye		*P* Value
**SCP, %**	Exotropia group	Control group		Exotropia group	Control Group	
Fovea	49.34 (2.57)	49.94 (2.67)	0.324	40.43 (2.36)	43.46 (2.43)	0.430
Inner macular	51.31 (3.01)	51.86 (3.36)	0.306	51.48 (3.43)	50.92 (3.21)	0.493
Temporal	51.57 (3.02)	51,79 (3.93)	0.415	49.49 (4.04)	51.03 (4.10)	0.839
Superior	52.20 (3.34)	52.52 (4.51)	0.724	52.35 (3.69)	51.72 (3.80)	0.369
Nasal	50.55 (3.75)	51.34 (4.44)	0.173	50.29 (3.93)	50.26 (4.71)	0.420
Inferior	50.94 (3.88)	51.77 (3.45)	0.365	51.41 (3.86)	50.65 (3.61)	0.407
**DCP, %**						
Fovea	37.08 (2.20)	36.92 (2.44)	0.796	36.38 (2.12)	35.92 (2.21)	0.932
Inner macular	**52.98 (3.85)**	**55.38 (3.51)**	0.007*	53.57 (4.56)	54.65 (3.59)	0.415
Temporal	51.57 (3.02)	56.97 (3.37)	0.014	55.15 (4.53)	56.07 (3.39)	0.362
Superior	52.75 (4.22)	54.00 (3.83)	0.211	53.36 (4.92)	53.40 (4.06)	0.797
Nasal	54.18 (4.12)	56.43 (4.21)	0.028	55.28 (3.86)	55.94 (3.73)	0.440
Inferior	51.22 (4.93)	54.14 (3.90)	0.013	52.30 (4.66)	53.21 (4.43)	0.554
**FAZ, mm**^**2**^	0.27 (0.09)	0.29 (0.06)	0.338	0.28 (0.09)	0.28 (0.08)	0.997
**RT, μm**						
Fovea	248.88 (17.67)	249.44 (17.92)	0.416	245.42 (17.07)	247.62 (16.76)	0.154

Data were expressed as mean (standard deviation)

*RT* Retinal thickness, *SCP* Superficial capillary plexus, *DCP* Deep capillary plexus, *FAZ* Foveal avascular zone, *SD* Standard deviation

* *p* value less than 0.01

Table 3 compares RT, macular vessel density, and FAZ parameters between the two eyes in strabismic subjects. The nasal quadrant RT was lower in the strabismic eyes than in the dominant eyes (330.60 [14.70] μm vs 332.88 [15.08] μm, *p* = 0.023). We found no significant differences in SCP or DCP (*p* > 0.01). The FAZ was not statistically different between the two eyes in exotropia subjects (*p* > 0.01).

**Table 3:** Retinal and macular microvasculature differences between deviated and dominant eyes in strabismic subjects

Variables	Deviated eye	Dominant eye	Difference	95%CI	*P* Value
**RT, μm**					
Fovea	248.88 (17.67)	245.42 (17.07)	1.12	(-1.66, 3.89)	0.425
**SCP, %**					
Fovea	43.11 (4.53)	43.43 (5.63)	0.23	(-1.59, 2.04)	0.804
Inner macular	51.31 (3.01)	51.48 (3.43)	-0.21	(-0.92, 0.49)	0.550
Temporal	51.57 (3.02)	49.49 (3.56)	-1.62	(-3.20, 0.05)	0.043
Superior	52.20 (3.34)	52.35 (3.69)	-0.18	(-1.03, 0.68)	0.683
Nasal	50.55 (3.75)	50.29 (4.20)	-0.54	(-1.92, 0.83)	0.433
Inferior	50.94 (3.88)	51.41 (3.86)	-0.08	(-1.00, 0.84)	0.865
**DCP,%**					
Fovea	36.37 (4.90)	37.08 (5.48)	0.81	(-0.75, 2.36)	0.304
Inner macular	52.98 (3.85)	53.57 (4.56)	0.14	(-0.90, 1.18)	0.790
Temporal	51.57 (3.02)	55.15 (4.53)	0.60	(-0.47, 1.66)	0.266
Superior	52.75 (4.22)	53.36 (4.92)	0.19	(-0.91, 1.30)	0.728
Nasal	54.18 (4.12)	55.28 (3.86)	0.56	(-0.49, 1.61)	0.296
Inferior	51.22 (4.93)	52.30 (5.06)	0.39	(-0.78, 1.56)	0.509

Data are expressed as mean (standard deviation)

*RT* Retinal thickness, *SCP* Superficial capillary plexus, *DCP* Deep capillary plexus, *FAZ* Foveal avascular zone, *SD* Standard deviation

## Discussion

In this study, we found reduced inner macular DCP in the exotropia group with abnormal binocular vision compared to the control group. We speculate that changes in the DCP may be associated with temporal hemiretinal suppression in the deviated eyes of exotropia subjects [20]. Visualization of neuronal activity in participants diagnosed with strabismus. To some extent, researchers have shown that the pathogenesis of strabismus may be related to changes in neuronal activity [15]. Retinal ganglion cells may be affected by efferent neuronal degeneration that originates in the visual pathway responsible for adaptation to the visual experience and atrophy of cells in the rostral superior colliculus region in the brain. Macular vascular density decreased after strabismus surgery, offering a new perspective on strabismus in the future [21, 22]. The decreased DCP was believed to participate in neuronal and vascular trophic factor reduction [23]. In addition, macular ischemia is associated with photoreceptor structural abnormalities in the visual pathway [24]. For other diseases including normal tension glaucoma and high myopia, reduced DCP was also found to be associated with retinal ischemia, oxygen insufficiency, thinner RNFL, and altered neuron RPE cells [23–28]. This may account for the abnormal visual pathway connection in strabismic subjects.

Previous studies have shown that the macular structure and retinal nerve fiber layer (RNFL) thickness were significantly altered in strabismic patients compared to healthy controls using OCT [15, 16, 22, 29–31]. Mintz. et al. [15] examined the retinal structure of 30 strabismic subjects after strabismus surgery using OCT. They showed that foveal and perifoveal RT was significantly greater postoperatively than that preoperatively (201.63 [18.36] µm vs 206.03 [22.73] µm, *p* = 0.024, 220.10 [16.23] µm vs. 225.80 [14.78] µm, *p* = 0.009, respectively). No differences were observed in the control eyes. This may contribute to the postoperative shift of extraocular muscle inflammation and the alternation of the blood-retinal barrier. Interestingly, Wen et al. [31] analyzed 138 different types of strabismic and healthy subjects using OCT and showed that the nasal quadrant in the inner macula and the temporal quadrant in the outer macula of RT were significantly increased in the concomitant and constant exotropia groups, respectively.

With the proliferation of medical imaging, OCTA directly manifests a more detailed visualization of retinal morphology and quantification of morphological microvascular metrics. As a non-invasive and effective examination tool, it sheds new light on the investigation of the sequelae of strabismus. In this study, we used OCTA to analyze the RT and capillary plexus in the exotropia group and the control group. We also investigated the difference between the strabismic eyes and the contralateral eyes in the exotropia group. We found that RT had no significant difference, which contributed to the similar SE and AL. This was consistent with the study conducted by Ji H. et al. [29] who compared the retinal thickness in 80 pediatric patients with intermittent strabismus. They found no difference in RT between mild, moderate, and severe strabismus. They also found no correlation between RNFL thickness and the onset of strabismus.

Reche-Sainz JA et al. [32] also showed that no statistically significant differences in RNFL thickness were found in any of these statistical comparisons using optical coherence tomography. They evaluated possible differences among children with strabismus and 32 controls, 31 esotropia, and 17 exotropia cases, and comparisons between dominant eyes and non-dominant eyes. They found no evidence that changes in RNFL thickness were associated with the presence of strabismus.

Compared with the control group, we found that in the exotropia group, SCP was greater in the temporal quadrant in the deviated eyes, and lower in the nasal quadrant in the dominant eyes in the outer macula. The previous reports were almost inconsistent [30, 33]. Li-Ying L. et al. [30] analyzed 29 constant strabismic patients and compared the macular microvasculature with age-matched healthy controls. They found no significant difference in RT, SCP, and DCP between dominant eyes, non-dominant eyes, and control eyes. This may be due to their small sample size, different statistical methods, and ethnic regions. We also examined DCP vessel density, which was lower in all quadrants except the superior quadrant. Some previous studies showed similar results. Zhai J. et al. [33] studied 40 patients with exotropia by OCTA in comparison with 36 control subjects. The macular perfusion density of the DCP in the non-dominant eyes of the patients with constant exotropia and those with intermittent exotropia was significantly lower than that of the control subjects. They identified macular microvasculature changes due to retinal ischemia or hypoxic injury. Regarding RT in the exotropia group, this study showed that the nasal outer macular was thinner than in control eyes.

Oka, M. et al. [16] analyzed patients with strabismus using OCT. They found that the superior to the inferior ratio of the macular RT and the temporal retinal ganglion cell complex was significantly lower in the strabismus group, which is different from what we have analyzed. It may be due to the degeneration of nerve cells in the binocular visual pathway. Li-Ying L et al. [30] reported that macular and temporal quadrant retinal thickness was significantly thinner in non-dominant eyes compared to dominant eyes (248.61 [19.84] μm vs 251.61 [19.37] μm, and 320.44 [17.05] μm vs. 323.44 [15.82] μm], *p* = 0.018, *p* = 0.018, respectively). Regarding the vessel density values in the deviated eyes, we found that the temporal quadrant SCP of the inner macular decreased in the deviated eyes compared to the control eyes concerning vessel density values. The analysis was consistent with the previous study by Zhai J.et al. [33] They reported that OCTA showed lower macular perfusion density in deviating eyes than in fixating eyes (47.1% [1.9%] vs 48.8% [2.2%], *p* = 0.032). Conversely, they showed that the macular perfusion density of the DCP in the 6*6-mm scan was lower in the deviating eyes than in the fixating eyes of patients with constant exotropia (*p* < 0.001, *p* = 0.032, respectively). The result was inconsistent due to the severe exotropia of patients with subnormal binocular monocular vision. This may reflect a chronic and degenerative process leading to vessel damage and disruption of the ellipsoid zone. The reduced flow signal of the SCP and DCP in acute intermediate maculopathy was identified by Chu et al. [34], suggesting that the reduced microvasculature is associated with ischemia of the macular area.

Furthermore, Yeo, J. H. et al. [35] investigated the microvascular changes in eyes with lamellar macular holes using OCTA and reported that better BCVA was associated with greater foveal vessel density in the SCP and DCP (*p* = 0.004, *p* = 0.005), and parafoveal vessel density in the SCP (*p* = 0.006), indirectly showing that the vessel density changes are related to the microvascular reduction. This may lead to visual impairment due to photoreceptor dysfunction. There was a statistically significant increase in vessel density measurements of the SCP and DCP, and a statistically significant decrease in measurements of the FAZ postoperatively (*p* < 0.05). However, there was no significant difference in FAZ parameters between the two groups, which is consistent with recent studies [30, 33].

### Strengths and limitations

To the best of our knowledge, this was the first study to investigate RT and macular microvasculature in patients with large-angle deviation whose binocular vision was abnormal in comparison with healthy controls based on OCTA. In addition, we analysed nine zones of macular values from the perspective of the deviated eye and the dominant eye. More importantly, we investigated the difference between the deviated eye and the contralateral eye in large-angle exotropia to explore the retinal morphological alternation and the possible pathogenesis of strabismus. In addition, we set a more stringent p-value of less than 0.01 to reduce the bias of comparisons.

It should be noted that our study had some limitations. First and foremost, a relevant small sample size was included in the analysis, which may introduce a little bias. The comparisons for various metrics inevitably caused statistical error. Therefore, we established standard inclusion and exclusion criteria to avoid possible errors, such as excluding those with poor control (NCS ≥ 4) and considering a significant p-value less than 0.01. Then, all subjects had a large exodeviation and required strabismus surgery, whether the remaining binocular vision alters the results is still unclear. In addition, patients with concomitant strabismus may not be attentive to the blue spot in OCTA imaging. Due to the limited economic condition of the hospital, we could not provide an ultra-widefield fluorescein angiography image. The 6*6-mm scan of the macular area may also be sufficient for basic identification. This may be one of the reasons for data bias. Further studies with a larger sample size are needed, including the evaluation of the macular vasculature and binocular vision to provide identical guidance for clinical diagnosis.

## Conclusions

In conclusion, patients with concomitant exotropia and abnormal binocular function have reduced temporal SCP in the dominant eye, nasal SCP, inferior, nasal, temporal SCP, and DCP in the strabismic eye. Changes in the macular microvasculature in exotropia may provide new insights into the stimulation of binocular vision. Further studies are needed to investigate whether reduced vessel density contributes to the pathogenesis of strabismus.

## Data Availability

The data used and analyzed in this study are available from the corresponding author.
